# Grassroots development of interprofessional primary care teams: a qualitative study in Canadian family practices

**DOI:** 10.3399/BJGP.2024.0756

**Published:** 2025-09-23

**Authors:** Asiana Elma, Aimun Qadeer Shah, Hila Shnitzer, Laurie Yang, Augustine Okoh, Michelle Howard, Jose Francois, Alan Katz, David Price, Lawrence Grierson

**Affiliations:** 1 research coordinator, Department of Family Medicine, Faculty of Health Sciences, McMaster University, Hamilton, Canada; doctoral student, Institute of Health Policy, Management, and Evaluation, Dalla Lana School of Public Health, University of Toronto, Toronto, Canada; 2 Department of Family Medicine, Faculty of Health Sciences, McMaster University, Hamilton, Canada; 3 Department of Family Medicine Max Rady College of Medicine, University of Manitoba, Winnipeg, Canada; 4 Department of Family Medicine, Faculty of Health Sciences, McMaster University, Hamilton, Canada; founding dean, SFU School of Medicine, Simon Fraser University, Surrey, Canada

**Keywords:** family practice, group practice, patient care team, primary health care

## Abstract

**Background:**

Primary care access is universally a critical health system concern. Decades of research shows that continuous, comprehensive, team-based care promotes greater access to promotional, preventive, and therapeutic care. In Canada, health system investments in team-based primary care models have not yet supported consistent implementation and uptake across the country. As a result, some family practices have taken a grassroots approach to realising team-based care.

**Aim:**

To explore the factors and processes that support the successful grassroots development of team-based family practices.

**Design and setting:**

Using a qualitative multiple case study design, we investigated the experiences of Canadian family practices that engaged in self-initiated efforts to develop or transform into a team-based care model in Canada.

**Method:**

Case-relevant documents and interviews with practice leaders were analysed using an unconstrained approach to qualitative description. Data collection and analyses were guided by the theory of social innovation.

**Results:**

Transformation processes were complex and multifaceted. Common activities across all cases were: developing business cases; obtaining funding; collaborating with provincial or regional governments, health authorities, and community members; ensuring buy-in from practice members; and securing space and human resources. These efforts supported alignment with local healthcare needs. Practice leaders uniformly declared that the change fostered positive outcomes, including improved access and attachment, more efficient workflows, and reduced emergency department visits.

**Conclusion:**

Those interested in promoting team-based family medicine should advocate for a balance of government investment and practice-level autonomy over development, which supports provider buy-in and community-appropriate innovation and responsiveness in care delivery.

## How this fits

Research has established that team-based care models improve access, continuity, and efficiency in primary care. Although much of the literature and system reforms have focused on government-led efforts in implementing team-based care models, little is known about the grassroots or practice-initiated approaches to implementing these models. This study offers novel understanding of the mechanisms by which family physicians and practice leaders enable self-initiated transformations, offering critical insights into the factors, processes, and, notably, the tensions that arise when self-initiated efforts aim to address systemic challenges within the constraints of the broader healthcare system. The findings provide actionable guidance for decision- and policymakers, health-systems leaders, and family physicians to support advancing context-specific team-based care models that align with community needs.

## Introduction

Health systems that include primary care that emphasises accessibility, continuity, and comprehensiveness achieve better health outcomes, greater health equity, and lower system costs.^1^ In Canada, primary care is a healthcare cornerstone, providing patients with ongoing care, preventive medicine, and a means to access secondary and tertiary care.^1,2^ Family physicians play a crucial role in this,^3^ diagnosing and treating health conditions, managing chronic diseases, coordinating with specialists and community, and conducting follow-ups. Despite this, more than 6.5 million Canadians are unable to secure a family doctor, leading to delays in diagnoses and treatment.^4,5^ The situation is expected to worsen as many family physicians are moving away from comprehensive care, reducing their clinical hours, or retiring; they cite untenable administrative burden and burnout as reasons.^6–8^

Government efforts to address access challenges were introduced in the early 2000s, leading to the establishment of team-based family medicine models such as family health teams in the Province of Ontario and family medicine groups in the Province of Quebec.^9–11^ By optimising the roles of healthcare professionals according to their expertise, team-based care improves care quality, relational continuity, resource utilisation, system efficiency, and provider job satisfaction.^12^ Indeed, considerable research has demonstrated the effectiveness of these models.^13–18^ Nevertheless, the implementation of team-based care remains fragmented, with several initiatives across the country facing inadequate infrastructure and insufficient financial support.^10,19,20^

In the absence of sufficient systemic support, some family physicians have self-initiated transformations to incorporate interprofessional teams and other key elements needed for a team-based care model. Although our understanding of these grassroots developments is nascent, recent research suggests that these team-based practices are as or more effective than those built through health authority initiatives in improving access, patient attachment, and the delivery of comprehensive continuity-based care.^18^ This may be because these transformations are better tailored to the needs and preferences of local communities. As the Canadian government begins to, once again, prioritise investment in the development of interprofessional primary care teams,^21–23^ we speculate that these self-initiated transformations can provide valuable insight into how teams can be built to improve local access.^24^ The aim of the study was therefore to better understand the factors and mechanisms that contributed to their successful development.

## Method

### Study design

Through a pragmatic constructivist approach, we employed multiple case study methodology to explore the grassroots development of team-based family medicine practices in Canada. Given the complexity of practice organisation and transformation,^25^ this methodology allowed us to build an understanding of the specific contextual factors and processes that supported the developments.^26^ Multiple cases allow for comparison within and across the practices.^26,27^ We anchored our work in the Theory of Social Innovation, which describes how social phenomena give way to new processes that redefine the routines, authority, and flow of resources within a social system.^28,29^ Accordingly, we considered the current system constraints on primary care access as a driver towards a more sustainable form of practice.^30,31^

### Case selection and boundaries

We leveraged previous research,^20^ professional networks, regional and provincial representatives, news and media reports, and relationships with the College of Family Physicians of Canada to identify family medicine clinics that had transitioned, in a grassroots way, to deliver interprofessional team-based care.^26^ These practices were deemed as ‘cases’ for this study.

A team-based transformation was characterised as grassroots if it was initiated by leaders and/or members of the practice and/or its local community, rather than under the auspices of a formal programme governed by a relevant regional health authority. A transformation that received support or contribution from the regional health authority would still be designated as grassroots provided the authority’s input was initially pursued by the practice. We sought practices that had recently transitioned (between 2014 and 2023) but were open to including any practice that had made such a transition since 1995.

We characterised a primary care team as interprofessional provided it included any mix and number of allied healthcare professionals working with family physicians to deliver primary care to a population, as long as the family physician served as the most responsible provider.^32^

### Participants

Eligible participants were family physicians and/or practice or administrative leaders who were actively involved in the case’s development or transformation process. These individuals were ideally situated to provide detailed information regarding the nature and characteristics of the practice and a historical perspective on the development process. Prospective participants were invited to participate in a semi-structured interview.

### Data collection

Semi-structured interviews (1 hour per interview) with eligible participants were held between June 2023 and October 2023. Interviews were conducted by five members of the research team trained in qualitative interviewing and with no prior relationship with participants. Interviewers included research staff (the first and second authors), medical students (the third and four authors), and a health system research scientist (the senior author). The interview guide was informed by literature and input from clinician (the seventh, eighth, and ninth authors) and health system research scientist (the sixth and the senior authors) collaborators.

The guide was piloted with a health system leader and was iteratively refined in consultation with the research team. Interviewers asked participants to describe the journey of developing or transforming the practice, with probes enquiring about the imperative for the transformation, the specific changes implemented, people involved, processes related to funding and resource acquisition, training and onboarding, and general change management. Following the interview, the interviewer generated a memo, highlighting key insights from the discussion.

The research team used the memos, coupled with analytical conversations every 2 weeks, to advance the analytic framing. This reflexive process often prompted new ideas that were explored in subsequent interviews. Interviews were recorded, transcribed verbatim, and de-identified before analysis.

Where relevant, we also reviewed case-specific reports and documents. These were not analysed directly but were used to provide case-relevant nuance and guide the nature and focus of interviews. Data collection was concluded on reaching data saturation.^33^

### Data analysis

We used an unconstrained approach^34^ to qualitative description^35^ while remaining attuned to the Theory of Social Innovation. First, we generated case descriptions of practice characteristics, before progressing to describing key patterns associated with practice development or transformation within and across cases. Two authors (the first and the senior authors) independently appraised four transcripts to form an initial analytic framework. This framework was then operationalised by other team members (the second, third, fourth, and fifth authors) on a randomly assigned sample of interview transcripts. To maintain rigour and consistency, all transcripts were double coded and managed in NVivo 14. The team held meetings every 2 weeks to review results, refine themes, and resolve coding discrepancies. This process culminated with a construction of results that addressed the research question.

## Results

Twenty-one practice leaders (Table 1), associated with 17 community-based practice cases (Table 2), participated in the interviews. For most cases, practice transitions had occurred relatively recently (between 2010 and 2022); however, five cases had transitioned earlier (between 1998 and 2009).

**Table 1.** Participant demographic characteristics (*N* = 21)

Participant characteristics	Value^a^
**Age, years, mean (SD)**	48.75 (8.85)
**Gender**	
Women	10
Men	11
**Role**	
Clinical leadership^b^	7
Practice manager	1
Physician member or associate	6
Executive leadership^c^	7

a *Data are *N* unless otherwise indicated.* ^*b*^ *Included medical lead, clinical director, chief medical officer, physician owner.* ^*c*^ *Included senior director, co-director, vice president, vice chair, president.*

**Table 2.** Aggregated locations, operations, and clinical attributes of cases (*N* = 17)

Case characteristics	Cases, *n* (%)
**Province**	
Alberta	2 (11.8)
British Columbia	2 (11.8)
Manitoba	4 (23.5)
New Brunswick	1 (5.9)
Newfoundland & Labrador	0 (0)
Nova Scotia	1 (5.9)
Northwest Territories	1 (5.9)
Nunavut	0 (0)
Ontario	4 (23.5)
Prince Edward Island	0 (0)
Quebec	0 (0)
Saskatchewan	2 (11.8)
Yukon	0 (0)
**Type of site**	
Teaching	11 (64.7)
Community	4 (23.5)
Unknown	2 (11.8)
**Distance to nearest hospital**	
In the same building	2 (11.8)
<5 km	8 (47.1)
5–20 km	3 (17.6)
>20 km	2 (11.8)
Unknown	2 (11.8)
**Geographic location**	
Rural	6 (35.3)
Urban	11 (64.7)
**Patient rostering**	
Yes	12 (70.6)
Average number of patients	
<500	1
500–999	5
1000–1500	5
*>*1500	1
No	2 (11.8)
Unknown	3 (17.6)
**Remuneration**	
Fee for service	3 (17.6)
Salary	5 (29.4)
Capitation	1 (5.9)
Service contract	2 (11.8)
Blended	4 (23.5)
Unknown	2 (11.8)
**After-hours clinic**	
Yes	10 (58.8)
No	5 (29.4)
Unknown	2 (11.8)

### Teams composition and organisation

Our participants described the diverse composition and organisation of their teams, which ranged from small groups with between one and four family physicians to larger teams of over 15 physicians, including part-time ones and/or physician associates. All teams reported including a range of nursing professionals. Most included health professionals such as pharmacists, mental health counsellors, and behavioural health consultants, with some also involving dietitians, social workers, and physiotherapists. Less common roles included respiratory therapists, phlebotomists, holistic wellness advisors, and audiologists. Administrative support was perceived as universally essential.

Participants described their practices as one of two team structures:

- *'*embedded' where interprofessional team members were integrated within the practice, working closely with family physicians to extend their care scope; or
- 'adjacent' where team members, often funded externally, collaborated from outside the practice while managing their own patient caseloads.

### Recognising and responding to the need for change

Prompting all transformations was the common realisation that the status quo was no longer sustainable to meet community needs. Increasing patient complexity, high morbidity, underserved needs, practice closures, physician retirement, and longer wait-times for patients to see a family physician were unanimously described as hindering local access to essential primary care services:

*‘It was about trying to bring awareness to a problem with primary care in* [city]*, and it was because we had four family practices, and one had just closed and there was quite an outcry about leaving so many people without a family doctor.’* (Participant 3)

All of our participants perceived team-based collaborative care to be an effective solution:

*‘What’s the solution? It is an interdisciplinary model. Change the patient’s thought of just being dependent on a person to be dependent on a team. If you can’t see me, that’s fine. You can see my team member. I can assure you you’ll get the same quality of care you get from me.’* (Participant 21)

### Common ingredients of successful developments

Participants described a common set of components that enabled their transitions. These included a dedicated champion, adequate funding, space, and technology, and support from relevant local and system-level partners.

#### A dedicated champion

Across all cases, participants identified family physician practice champions who spearheaded the transformation process. Although the participant being interviewed was often the champion, in some cases, particularly when the transformation had taken place long ago, the described champion was someone that was holding a leadership position at the time of the change. These influential, passionate, and committed ‘champions’ were viewed as visionaries and key decision-makers within their practices and the broader community. They were recognised for their strong ties with influential figures across the healthcare system. These extensive professional networks were reportedly cultivated through years of leadership within the system. In leveraging these connections, the champions coordinated efforts by bringing together diverse partner groups, and effectively communicated the mission and vision of the transformation to ensure alignment and collaboration among all involved:

*'Under their direction and the passion that they had, there was lots of interdisciplinary groups coming forward … because of that leadership that they had and the passion that they had, it actually solidified into disciplinary things and great patient outcomes came from it.'* (Participant 5)

#### Partner involvement

Our participants strongly emphasised the essential value of partner collaboration, describing active engagement with local communities in co-design and implementation efforts, including patients, system leaders, and others. Engagement with practice members was seen as critical for identifying challenges within the local context. Equally important was the collaboration with regional or provincial government for strategic planning and navigating complex bureaucratic systems. Comprehensive engagement across these levels was instrumental in supporting transitions, ensuring initiatives were well-rounded, informed, and tailored to address prevailing local needs:

*‘*[I]*t was a huge co-design initiative made up of partners, experts, physicians, patients, to really look at how do we structure a model that is going to work in this region. And that took about two years of work before this site even opened, and a lot of leadership at various levels of different organisations*.*’* (Participant 14)

#### Adequate funding

Practices acquired the necessary financial resources through a combination of various funding corridors including self, community, and government investment. Regardless of the mix, funding supported salaries for health and administrative professionals, physical spaces, electronic medical record systems, and other operational requirements.

Participants across approximately one-third of cases reported self-investing in their practice, using practice-generated revenue, personal reserves, or bank loans to fund their team-based transition. These participants espoused an entrepreneurial stance, and expressed a sense of responsibility in investing in their practices to meet community needs:

*‘GP has to go and reflect on how we do things. GP has to take chances. GP has to spend money … in a project and that’s all life changing.’* (Participant 1)

Many participants described receiving community funding through donations and investments from local organisations, foundations, and municipalities:

*‘*[T]*he community came together … the community needed to raise an extra one million dollars to have the physician’s office attached. That was definitely cut by the* [Health Authority]*. That’s what happened is the community raised that money because they saw that that was the most practical*.*’* (Participant 5)

Although transformations were not explicitly mandated by health authorities, participants uniformly described securing some measure of government support. Most described initiating the transformation independent of any government involvement. However, as the need for resources became apparent, they lobbied for resources and/or applied to established funding programmes offered by provincial, territorial, or regional authorities. In a small number of cases, participants described recognising a prominent government initiative, such as a pilot programme, that they sought to facilitate the change:

*‘It started as a shared care pilot. So back in* [province] *there were six shared pilots, and what really started to get me into a team was the fact that the government had given some funds for us to add allied health professionals to a practice of three physicians*.*’* (Participant 19)

However, government funding was sometimes described to come at a cost. Although most practice leaders retained control over the transformation, those that received the greatest government investment often described new requirements to meet government-set performance measures that raised apprehensions about durable practice autonomy:

*‘I think there was some fear of losing autonomy … are they going to start to tell me what to do and how to operate my practice?’* (Participant 14)

#### Space and technology

Transitioning into a team-based practice also required the acquisition of new physical space and supportive technological infrastructure. This was consistently described as essential for accommodating additional team members, facilitating effective workflows and communication, and supporting collaboration:

*‘*[W]*e co-located the teams to sit together. There’s a fair bit of on-the-fly personal communication, particularly between the docs and the nurse and between the docs because the docs and the nurses sit together and if we're dealing with a complicated patient sometimes it’s through the EMR* [electronic medical record]*.’* (Participant 11)

Technology that facilitated team connection and communication was described as particularly useful in cases where members of the team were not co-located in the same space.

### Common processes of successful developments

The process of transformation was characterised as sequential stages — gaining an understanding of local needs, building a business case, managing the change, and committing to continuous quality improvement. At each stage, a range of challenges, facilitators, and barriers were at play, each of which had an impact on the change trajectory.

#### Determine local needs

Practice leaders recognised the importance of aligning the composition of their team with the specific needs of the local community. This process involved reviewing national population reports and engaging with local community to gain insights into patient demographics, prevalent health issues, and other determinants of health. The identification of community needs was described as ‘dynamic’ and involved comparisons with other successful team-based primary care practices: regionally, nationally, and internationally:

*‘*[I]*f I want to … add a different kind of person to my team … you need to do your homework, you’d start with looking at national standards around whatever the issue might be, and then you start talking with managers of the clinic … other people in your team*.’ (Participant 2)

In some cases, independently contracted organisations conducted needs assessments and developed strategic plans.

#### Make a business proposal

With community needs identified, participants reported moving forward with the development of compelling business proposals to support the transformation process. These detailed the current state of the practice, described the intended changes, and justified the need for additional resources. They were typically created by the practice champion in collaboration with local community or health system leaders:

*‘We hosted this massive community engagement with hundreds of people … We started a process of engaging the physicians to come along and said, here’s our proposal. What do you think the problems are? What do you think? We’d already done the outreach to all the stakeholders — our municipality, provincial government, local government, health authority. Then we really started doing outreach to the providers and family practice.’* (Participant 4)

Notably, the presentation of business cases to health authority partners was perceived to be onerous:

*‘The lobbying of the government takes up an enormous amount of time that’s unnecessary ... It’s beyond what is needed to put a case forward or a case example forward and that scares off a lot of physicians … It’s not a streamlined process, it’s not a user-friendly process*.*’* (Participant 19)

#### Manage the change

Participants described embarking on a progressive process of change management once the vision was articulated, resources were secured, and the plan was set. A key part of this involved inspiring ‘buy-in’ from the team. Participants stressed that team members needed to believe in and enjoy being part of a collaborative environment. However, achieving this level of engagement was not always easy. Practice champions often needed to facilitate a shift in thinking about care delivery before team engagement was fully realised:

*‘There’s still growing pains in the clinic. Every month we have a monthly operations meeting where the whole clinic gets together and talks about what can be improved … There’s still growing pains but overall, we’re making this work*.*’* (Participant 10)

Buy-in was achieved through training, regular communications, and the nurturing of a trusting work culture. Participants emphasised that teams needed to be trained on the principles of team-based care and new procedures to support this style of care delivery. This knowledge was shared via comprehensive communications and full team huddles, focusing on workflow changes and role adjustments. These engagements, however, went beyond training and education — they also served as important touchpoints for elevating team morale and nurturing team culture. Indeed, our participants highlighted how trust was an important facilitator, and that this was built through formal communications and workplace behaviours.

#### Improve continuously

A commitment to continuous quality improvement was crucial, including regular assessment, evaluation, and iterative adaptation. Insights were regularly gathered from patients, providers, partners, communities, and municipal leaders. Data-collection methods varied, including satisfaction surveys and informal feedback. In some cases, external organisations collected and analysed quality improvement data. In others, regional primary care networks managed the collection and analysis of quality improvement data. Government agencies conducted data collection and needs assessments within those practices with heavy government involvement. These activities enabled ongoing adjustments that improved effectiveness and efficiency:

*‘*[W]*e did surveys, we did interviews. We looked at numbers, we tried to look at data … one of our values is to be data driven* …*’* (Participant 4)

### Perceived impact and outcomes

Participants told us that their transitions had a profound impact on patients, their practice, and the healthcare system. Improvements in accessibility, scope of service, and community health outcomes were consistently mentioned, regardless of the level of government support received. Participants noted increased capacity to take on more patients and a new ability to leverage diverse expertise to offer comprehensive, well-coordinated care. Some participants also reported improvements in patient health outcomes:

*‘People were losing weight, blood pressures were down, their vitals were better. Everything was great, chronically better* …*’* (Participant 1)

For healthcare providers, the transformations led to greater efficiency and better workflows. These changes were perceived to enhance job satisfaction and reduce burnout. The collaborative environment also created valuable learning opportunities for family physicians:

*‘I can distinctly remember one of the family practice residents saying, “I didn't know you knew all that” … And the midwife looked at them and said, “This is what I do. I trained for four years in delivering and taking care of — ” And there was this ah-ha moment. It’s like, oh, yeah, you have this opportunity within your family practice for this much training in maternity care*.*’* (Participant 16)

At the system level, the transformation was described as improving the efficient use of healthcare services, such as a reduction in local emergency department visits:

*‘When the second to last family practice closed, our emergency department started tracking how many visits they were getting, particularly in low acuity situations … and the numbers just started going up and up and up. It was people showing up for UTIs* [urinary tract infections] *and prescription renewals and things that like really should have been dealt with by a family doctor … and then our lead medical director just shared some results. She was looking at year-over-year visits now for January, February, March … The increase has dropped and as of May is actually less than May last year. So, it’s like, “Oh wow, this is exactly what we're hoping for*.”*’* (Participant 3)

## Discussion

### Summary

This study illustrates that the grassroots development of interdisciplinary, team-based care practices is a complex and dynamic process, influenced by local healthcare needs, leadership, regional and provincial contexts, and the availability of adequate resources. These factors informed the mechanisms that enabled our participants to secure funding, foster collaborative relationships, navigate interpersonal dynamics, and commit to continuous quality improvement. However, the grassroots nature of these efforts also introduced tensions, as practices sought to balance their autonomy in driving the change process with the need to align with broader health system priorities and requirements. Nonetheless, the interplay between these factors and mechanisms was essential to ensure that transformations aligned not only with the needs of patients, practice staff, and the broader healthcare system, but also to support ongoing evolution, enabling practices to adapt and meet needs as the healthcare landscape changes.

### Strengths and limitations

Our study covers a broad geographic range, including eight provinces and territories, and data collection continued until saturation was reached, capturing diverse processes in developing interprofessional primary care teams. The interview guide and coding framework were developed with input from health systems and clinical experts, and analysis was conducted by independent reviewers to ensure consistency. A limitation is that our study focused on participants who successfully implemented team-based care, which may not fully reflect the experiences of those facing challenges. Additionally, although participants highlighted the importance of perspectives from regional or municipal leaders, decision-makers, communities, and patients, these viewpoints were not included.

### Comparison with existing literature

The perspectives shared by our cases contribute to and expand on the existing body of research on team-based care by detailing the mechanisms and nuanced dynamics that practices navigate when developing and transforming care models. In this process, a clearly articulated vision for team-based care emerged as a critical enabler in our study, facilitating partner buy-in and promoting a cooperative transition process.^36^ This finding aligns with existing literature, which emphasises the importance of effective communication within the team and with external collaborators to align goals and foster shared decision-making.^37–40^ Additionally, change management was pivotal, with participants highlighting the need for regular team engagement and a collaborative culture to make the vision for team-based care actionable and navigate practice changes effectively.

However, these grassroots efforts introduced tensions as our participants independently initiated bottom-up transformations to address systemic issues of primary care access. Operating within a healthcare system designed to support centralised or government-led initiatives, these efforts often required balancing autonomy to innovate with system-level structures. Notably, this autonomy was highly valued by our participants, especially when designing community-specific solutions tailored to their unique contexts. In Canada, where family physicians operate as self-regulated professionals, this autonomy likely reinforces their inclination to innovate and adapt their practices through grassroots efforts. This reflects the challenge of balancing locally informed insights with system-level support. Although government investment provides structure for team-based care transformation, rigid top-down policies often misunderstand regional constraints, local needs, and existing processes, inadvertently exacerbating disparities instead of reducing them.^41^ This highlights the need for collaborative approaches that integrate the strengths of grassroots efforts with supportive system-level policies and structures.

Lastly, our findings emphasise engaging in continuous quality improvement processes to support successful transformations. However, barriers such as change fatigue, insufficient time, and the substantial in-kind work required from practice staff must be addressed. Allocating protected time for evaluation can help maintain staff satisfaction and alleviate burnout.^42^ Health authorities can support these efforts by implementing comprehensive monitoring systems to assess the impact of the transformation.^43,44^ Complementing these measures with educational initiatives that promote interprofessional collaboration is equally important. Without proper training, teams may face challenges such as disrupted care continuity, power imbalances, and compromised communication, which can negatively impact patient outcomes.^45,46^ Preparing healthcare teams to work together effectively is needed for the success of team-based care models.^47^

### Implications for research and practice

Given the complexity of grassroots transformations into team-based care models, this study has implications for primary care practices and key decision- and policymakers. For practices, incorporating perspectives of stakeholders, including health authorities, community members, and patients, should be considered to ensure initiatives align with their needs and expectations. Practice champions leading these efforts should strategically leverage political and social capital to secure the necessary support for the change, while aligning their initiative with regional and provincial priorities to foster collaboration and sustainability. Simultaneously, respective health authorities should provide primary care practices with the necessary resources to support locally driven innovations while maintaining practice autonomy, and streamline proposal processes to encourage bottom-up initiatives.

## Data

The semi-structured interview guide is available for review on request to the corresponding author.
